# Surface glycans contribute to differences between seminal prostasomes from normozoospermic and oligozoospermic men

**DOI:** 10.1080/03009734.2019.1592266

**Published:** 2019-04-08

**Authors:** Bojana Milutinović, Sanja Goč, Ninoslav Mitić, Maja Kosanović, Miroslava Janković

**Affiliations:** University of Belgrade, Institute for the Application of Nuclear Energy, INEP, Zemun, Serbia

**Keywords:** Glycosylation, prostasomes, seminal plasma, sialic acid

## Abstract

**Background:** Extracellular vesicles (EVs), released from the plasma membrane or intracellular compartments, have a specific composition related to their parent cells, but they can, additionally, be modified by the extracellular environment. Although glycans are known to contribute to EV composition and may have biomedical importance as biomarkers and recognition signals, they have not been extensively investigated. In this study, seminal prostasomes, i.e. EVs from seminal plasma (SP) of normo- and oligozoospermic men, were analyzed in order to detect possible changes in their surface glycans under altered physiological conditions.

**Methods:** Prostasomes were isolated from pooled SP by differential centrifugation and gel filtration, followed by glycobiochemical characterization using lectin/immune-transmission microscopy and ion-exchange chromatography.

**Results:** Within the frame of overall similarity in protein composition, surface glycans specifically contributed to the differences between the examined groups of prostasomes in terms of presentation of sialylated and mannosylated moieties. These changes did not affect their anti-oxidative capacity, but implied a possible influence on the accessibility of galectin-3 to its ligands on the prostasomal surface.

**Conclusions:** Subtle differences in the presentation of surface molecules may be helpful for differentiation among vesicles sharing the same physical properties. In addition, this may point to some unexpected regulatory mechanisms of interaction of distinct populations of vesicles with their binding partners.

## Introduction

Extracellular vesicles (EVs) are nano-sized membrane-enclosed structures released by all cells in the extracellular environment (1–4). EVs’ ability to carry molecules, to pass through biological fluids, cross barriers, and target specific cells provides them with a great potential to be used as next-generation natural nano-tools (5,6).

Diversity in the composition of lipids, proteins, and nucleic acids associated with EVs is related to parent cells or tissues, and, in this way, they act as specific biogenetic markers, at the same time carrying markers common to all EVs (7,8). However, it is possible that EVs could also be modified by the extracellular environment, but data concerning externally adsorbed components including glycoproteins are scarce (9). Thus, both influences pervade EVs’ composition, leading to the formation of a distinct molecular pattern of their luminal/internal cargo, the membrane itself, as well as their surface cargo (9). Some of these patterns were shown to be changed under different physiological and pathophysiological conditions (2) and, consequently, may have significant biomedical importance.

This study is a comparative analysis of EVs isolated from seminal plasma (SP) of normo- and oligozoospermic men, focused on their glycosylation. It is known that a majority of EVs in SP are prostasomes, EVs secreted by the prostate (10). They were originally examined in prostatic fluid and subsequently in SP (10–12). In contrast to accumulated experimental data on prostasomal ultrastructure and composition of proteins and lipids (10), glycans, i.e. complex oligosaccharides, have not yet been studied in detail. An altered glycoproteome of SP has already been observed and related to different sperm count and fertility status in comparison to SP obtained from ejaculate with normal parameters (13,14). However, few studies have addressed the diversity of prostasomes in related conditions. In general, quantitative rather than qualitative differences in the examined prostasome parameters were found in SP from infertile subjects or those with abnormal semen parameters in comparison to values for SP of healthy men. Thus, in one study, the number of vesicles and associated enzymatic activity was found to be very low in azoospermia (15). Differences in levels of expression of some common proteins were reported recently, most of which were overexpressed in prostasomes from normozoospermic men compared to those from non-normozoospermic (16).

Thus, we aimed at resolving potential differences in surface glycans of prostasomes from selected SP samples. As recognized components of lipids and proteins, surface glycans are presumed to contribute significantly to different functional activities of prostasomes. As in the case of cell–cell and cell–molecule interactions where carbohydrate recognition plays a crucial role, glycans on prostasomes could constitute molecular requirements for their action as a communication tool and harbor biomarker structures (17). According to biogenesis data, the prostasomal membrane is kindred to the plasma membrane of the ancestral epithelial cell (18) and, in this way, may be a reflection on the vesicle surface of an altered status of prostate physiology, as already proposed for reflection on vesicle cargo (16,18).

## Materials and methods

### Material

Monoclonal anti-CD63 antibody (clone TS63) was from Abcam (Cambridge, UK) and biotinylated goat anti-galectin-3 (gal-3) antibodies from R&D Systems (Minneapolis, MN, USA); 3,3′,5,5′-tetramethylbenzidine (TMB), bovine serum albumin (BSA), D-lactose, and methyl- alpha D-mannopyranoside were from Sigma (St. Louis, MO, USA). Biotinylated goat anti-mouse IgG, biotinylated plant lectins, concanavalin A (ConA), SNA (*Sambucus nigra* agglutinin), and the Elite Vectastain ABC kit were from Vector Laboratories (Burlingame, CA, USA). Sephadex G 200 and Sephadex DEAE A-50 were from Pharmacia AB, Uppsala, Sweden. The silver stain kit and SDS-PAGE molecular mass standards (broad range) were from Bio-Rad (Hercules, CA, USA). Nitrocellulose membrane and Pierce ECL Western Blotting Substrate were from Thermo Scientific (Rockford, IL, USA). BCA Protein Quantification Kit was from Abcam (Cambridge, UK). Microwell plates were from Thermo Scientific (Roskilde, Denmark). Lymphocyte separation medium LSM-B was from Capricorn Scientific GmbH (Ebsdorfergrund, Germany).

### Human semen samples

This study was performed on leftover, anonymized specimens of human semen taken for routine analysis, and since existing human specimens were used it is not considered research on human subjects. The study was approved by the institutional Ethics committee according to the guidelines (No.# 02-832/1), which conform with the Helsinki Declaration, 1975 (revised 2008). Sperm parameters were assessed according to the recommended criteria of the World Health Organization (released in 2010), concerning numbers, morphology, and motility. Sperm cells and other debris were removed from the ejaculate by centrifugation at 800 × *g* for 20 min.

### Isolation of prostasomes from human seminal plasma

Two pools of human SP of normozoospermic (N) and two pools of human SP of oligozoospermic (O) men were used for isolation of prostasomes. Each pool contained 10 individual seminal plasma samples. Prostasomes were isolated from SP according to a modified protocol of Carlsson et al. (19) in that CD63-immunoreactivity was used as an indicator of the presence of EVs.

### Ion-exchange chromatography (IEC) of seminal prostasomes

A DEAE Sephadex A-50 column (10 mL bed volume) equilibrated with 0.05 M Tris–HCl buffer, pH 7.6, was used for IEC of EVs as described before (20). Briefly, after loading of prostasomes (1 mL), non-bound material was washed away with equilibration buffer, followed by step-by-step elution with 0.05 M Tris–HCl buffer, pH 7.6, containing 0.05 M NaCl, 0.1 M NaCl, 0.2 M NaCl, and 1 M NaCl. Fractions (1 mL) were collected, immobilized on microwell plates, and subjected to a lectin-binding assay using ConA and SNA to monitor elution of charge-resolved populations (20). Initially, specificity of the lectin-binding was confirmed by inhibition using 0.2 M lactose (for SNA) and 0.2 M methyl- alpha D-mannopyranoside (for ConA).

### SDS-PAGE

Proteins were resolved on 10% separating gel with 4% stacking gel under denaturing and reducing conditions (21) and stained with silver nitrate, using a silver stain kit (Bio-Rad) according to the manufacturer’s instructions. The gel was calibrated with SDS-PAGE molecular weight standards (broad range).

### Western and dot blot

Samples were transferred onto nitrocellulose membrane by semi-dry blotting using a Trans-blot SD (Bio-Rad). The conditions were: transfer buffer, 0.025 M Tris containing 0.192 M glycine and 20% methanol, pH 8.3, under a constant current of 1.2 mA/cm^2^ for 1 h. The membrane was blocked with 3% bovine serum albumin (BSA) in 0.05 M phosphate buffer saline (PBS), pH 7.2, for 2 h at room temperature, and then incubated with anti-CD63 antibody as described below.

For dot blot, 3 µL of each corresponding fraction was applied to the nitrocellulose membrane, dried, and blocked as described above.

### Immunoblot analysis

For immunoblot analysis, proteins on the membrane were incubated with anti-CD63 antibody (20 μg/mL) overnight at 4 °C. After a washing step, bound antibody was detected by incubation with biotinylated goat anti-mouse IgG for 30 min at room temperature. The membrane was rinsed, and the HRPO mixture from the Elite Vectastain ABC kit was added. After another washing step the blots were visualized using Pierce ECL Western blotting substrate according to the manufacturer’s instructions.

### Transmission electron microscopy (TEM) and lectin-TEM

TEM was performed as described previously (20). For lectin-TEM, samples were applied to the Formvar-coated, 200-mesh, Cu grids by grid flotation on 10 μL sample droplets, for 45 min at room temperature. This was followed by steps of: fixation (2% paraformaldehyde, 10 min); washing (PBS, 3 × 2 min); quenching (0.05 M glycine, 10 min); blocking (2% BSA, 30 min); washing (0.1% BSA/PBS, 6 × 2 min); binding of either (a) biotinylated lectins (SNA, 0.4 μg/mL and ConA, 0.62 μg/mL, 1.5 h at room temperature) or (b) biotinylated anti-gal-3 IgG (0.5 μg/mL of 1% BSA/PBS, 1.5 h at room temperature); binding of streptavidin-gold (1:100 in 0.1% BSA/PBS, 1 h at room temperature); washing (0.1% BSA/PBS, 3 × 2 min); post-fixing (2% glutaraldehyde, 5 min); and final wash with dH_2_O. Grids were then air-dried and images were collected using a Philips CM12 electron microscope (Philips, Eindhoven, The Netherlands).

### Preparation of polymorphonuclear neutrophils (PMN) from whole blood

PMN were isolated from a healthy blood type O + donor. Whole blood was collected in EDTA-containing tubes and left to sediment for 2 h at room temperature. The supernatant and upper sediment were layered on 3 mL of lymphocyte separation medium and centrifuged at 1800 rpm for 30 min. The pellet, containing PMN and erythrocytes, was treated with isotonic 0.15 M NH_4_Cl containing 4 mM NaHCO_3_ lysing solution, pH 7.3, for 10 min on a rotating mixer. The residual neutrophils were washed in 0.05 M PBS, pH 7.2, and pelleted by centrifugation at 1800 rpm for 10 min. Isolated PMN were counted manually using a Neubauer chamber.

### Determination of reactive oxygen species (ROS) production in PMN

PMN activation assay was performed as previously described (22). Briefly, 50 µL of cell suspension (250,000 cells), 10 µL of the corresponding sample (total protein concentration 100 µg/mL), 10 µL of luminol, and 10 µL of phorbol myristate acetate (PMA) (40 µg/mL) were added. To monitor baseline ROS production, the samples were substituted with 0.05 M PBS, pH 7.2. Chemiluminescence was measured using a Wallac 1420 Multilabel counter (PerkinElmer, Monza, Italy) over 30 min with a 5-min counting delay and expressed as arbitrary units (AU). Results are presented as the mean value with standard deviation (SD) of three separate experiments. Statistical analysis was carried out using paired Student’s *t* test, with mean values considered significantly different when *P* < 0.05.

## Results

### Electron microscopy

**Figure 1.**
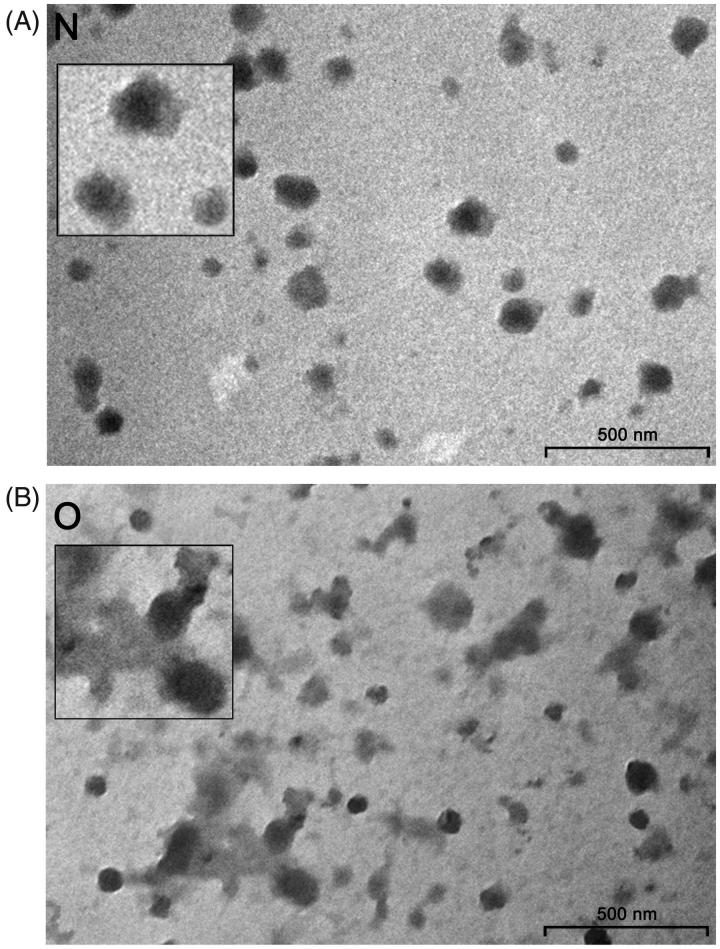
Transmission electron microscopy of seminal prostasomes. Inserts show enlarged the typical appearance of seminal prostasomes from normozoospermic men (N) (no protein in the background) and seminal prostasomes from oligozoospermic men (O) (protein material in the background with vesicles seemingly immersed).

There were light and dark vesicles ranging from 50 to 150 nm in both groups of samples. However, in contrast to seminal prostasomes of normozoospermic men (Figure 1(A)), those of oligozoospermic men contained more amorphous substance with one or several vesicles appearing immersed in them (Figure 1(B)).

### SDS-PAGE

**Figure 2.**
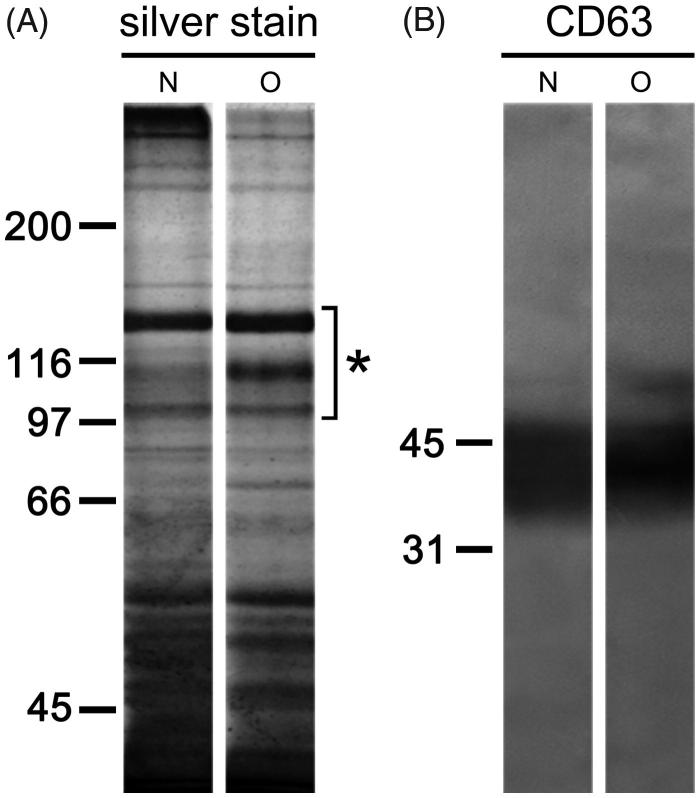
Protein composition of seminal prostasomes. Seminal prostasomes isolates were resolved on 10% SDS-PAGE under reducing and denaturing conditions and stained with silver or transferred onto a membrane and subjected to immuno-blot. A: A representative total protein pattern of seminal prostasomes with three characteristic bands (12) in the region of 90–150 kDa (asterisk). B: CD63, an EVs-associated marker gave characteristic smeared band above 31 kDa. The numbers indicate the position of molecular mass standards (kDa). (N = seminal prostasomes from normozoospermic men; O = seminal prostasomes from oligozoospermic men).

When isolated seminal prostasomes were resolved electrophoretically, complex patterns of differently abundant and partially overlapping protein bands ranging from 35 kDa to over 250 kDa were observed (Figure 2(A)). In addition, there was comparable immunoreactivity to CD63, the common marker for EVs (Figure 2(B)).

### IEC

**Figure 3.**
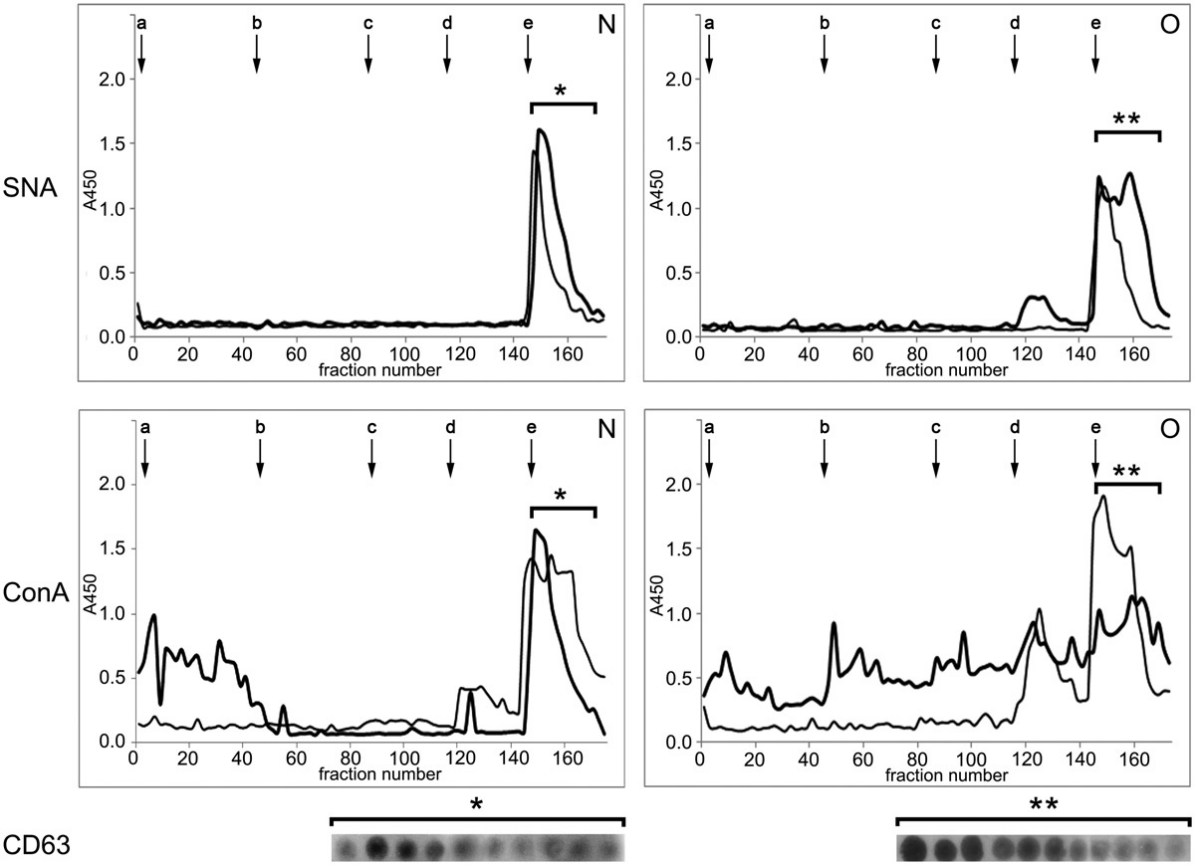
Surface glycosylation of seminal prostasomes: ion-exchange chromatography. Two pools of each group of isolates were subjected to ion-exchange chromatography on DEAE A-50 column eluted with 0.05 M Tris–HCl buffer, pH 7.6 (a), containing: 0.05 M NaCl (b); 0.1 M NaCl (c); 0.2 M NaCl (d), and 1 M NaCl (e). Elution was monitored by *Sambucus nigra* agglutinin (SNA) and concanavalin A agglutinin (ConA) binding reactivity. N pool 1 (thick line) and N pool 2 (thin line) had protein concentration 313 µg/mL and 473 µg/mL, respectively. O pool 1 (thick line) and O pool 2 (thin line) had protein concentrations of 318 µg/mL and 581 µg/mL, respectively. Asterisk denotes CD63-positive fractions as determined by dot blot. (N = seminal prostasomes from normozoospermic men; O = seminal prostasomes from oligozoospermic men).

The surface composition of examined seminal prostasomes was initially inspected by IEC (Figure 3). There were no striking differences within the samples. Thus, representative elution profiles of charge-resolved prostasomes of normozoospermic and those of oligozoospermic men, as detected by *Sambucus nigra* agglutinin and ConA, suggested a major population released with 1 M NaCl (constitutively present in all four pools) in both samples. It was positive for CD63 by dot blot, indicating a concentration of negatively charged vesicles in it. The SNA-binding pattern of a population of prostasomes from normozoospermic men eluted with 1 M NaCl revealed a relatively sharp peak exhibiting a hint of unseparated populations in its trailing edge, whereas at least two partially separated subpopulations were observed among prostasomes of oligozoospermic men. In contrast to this, ConA-binding patterns seemed to be more heterogeneous, appearing as several more or less separated subpopulations.

In addition to vesicles-enriched populations (CD63-positive), both SNA and ConA revealed lower-charge CD63-negative ones (occasionally present). In general, there was a broad elution profile across these populations, especially for prostasomes of oligozoospermic men. Thus, in prostasomes of oligozoospermic men, there were CD63-negative populations eluted in the flow-through fraction as well as with 0.05 M, 0.1 M, and 0.2 M NaCl. The population eluted by 0.2 M NaCl reacted with SNA and ConA, whereas the others reacted with ConA only. In prostasomes of normozoospermic men, one CD63-negative population was separated in the flow-through fraction, and it was ConA-reactive.

### Lectin-TEM

**Figure 4.**
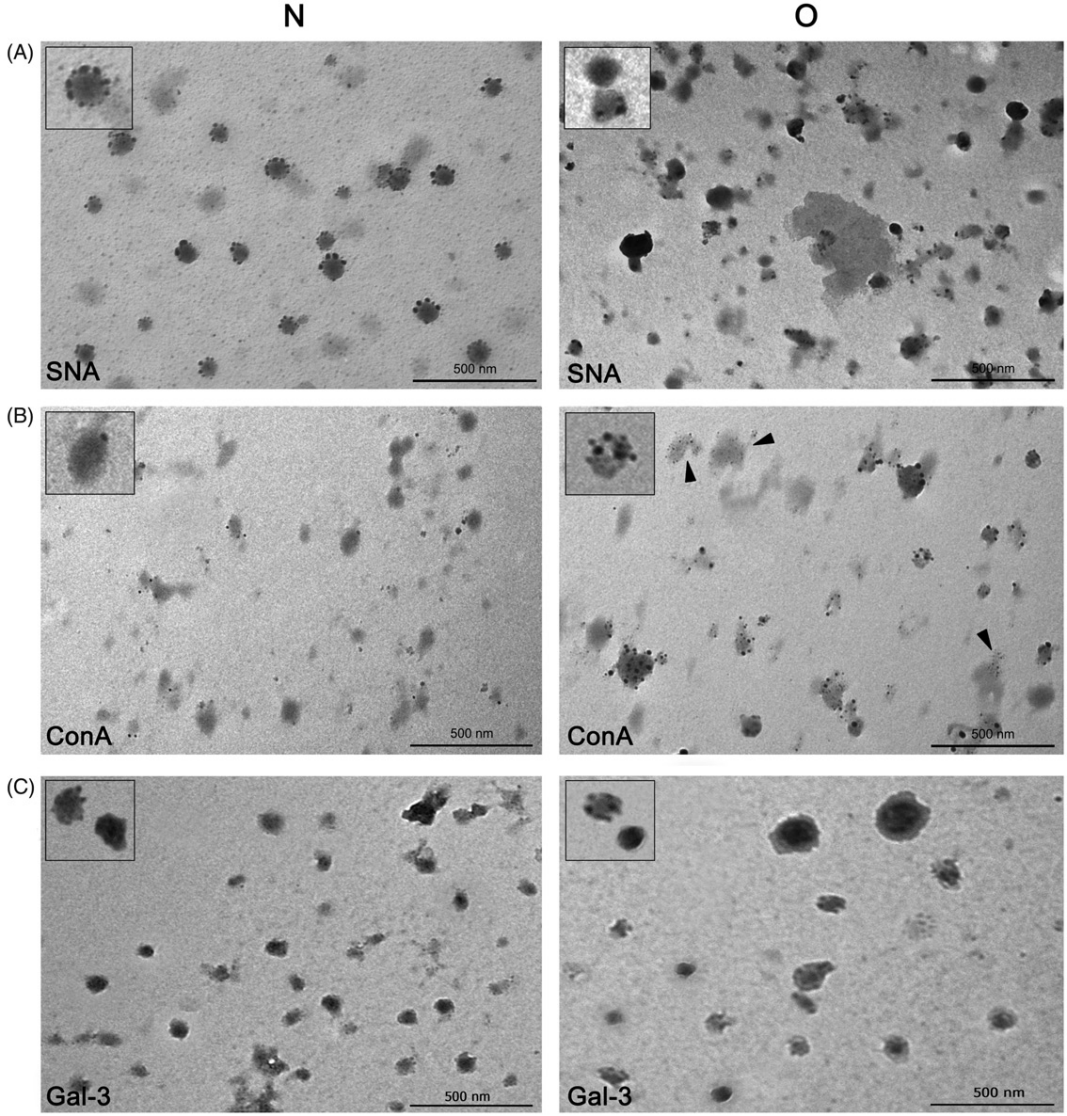
Surface glycosylation of seminal prostasomes: lectin- and immune-transmission electron microscopy. A: Lectin-TEM using SNA. Inserts show enlarged characteristic pattern of SNA-reactivity to each sample group. B: Lectin-TEM using ConA. Inserts show enlarged characteristic pattern of ConA-reactivity to vesicles in each sample group. In O, staining of some proteinaceous material was also observed (arrowheads). C: Immune-TEM using anti-galectin-3 antibodies. Inserts show enlarged characteristic pattern of anti-gal-3-reactivity to each sample group. Micrographs show most characteristic patterns obtained. Although differences in the reactivity of particular vesicles could be noticed, it does not affect the general reactivity of the sample (as in IEC when taking all vesicles into account). (N = seminal prostasomes from normozoospermic men; O = seminal prostasomes from oligozoospermic men).

The subtle differences implied by IEC between prostasomes of normozoospermic men and those of oligozoospermic men were further analyzed using lectin-TEM. Native samples were analyzed by means of both techniques, but, in contrast to IEC, which concentrates different vesicles according to their general negative charge, lectin-TEM is expected to give more insight into presumed heterogeneity among particular vesicles based on homogeneity/intensity of staining of selected glycans on their surface. In the preparation of prostasomes from normozoospermic men, SNA bound to all vesicles, having a rosette-like appearance, whereas not all prostasomes of oligozoospermic men were stained. In contrast to vesicles, no staining of surrounding proteins was observed (Figure 4(A)).

As for ConA only some vesicles were stained in the preparation of prostasomes of normozoospermic men (Figure 4(B)). In contrast to that, almost all vesicles in the preparation of prostasomes of oligozoospermic men were stained. In addition, in prostasomes of oligozoospermic men, there was staining of some amorphous material as well.

In the context of surface composition, the EVs marker gal-3 was also examined (Figure 4(C)). It was observed in both sample groups by immune-TEM, and there were no striking differences as regards its antigenic moieties in prostasomes of normozoospermic men compared to those of oligozoospermic men.

### ROS production in PMN

**Figure 5.**
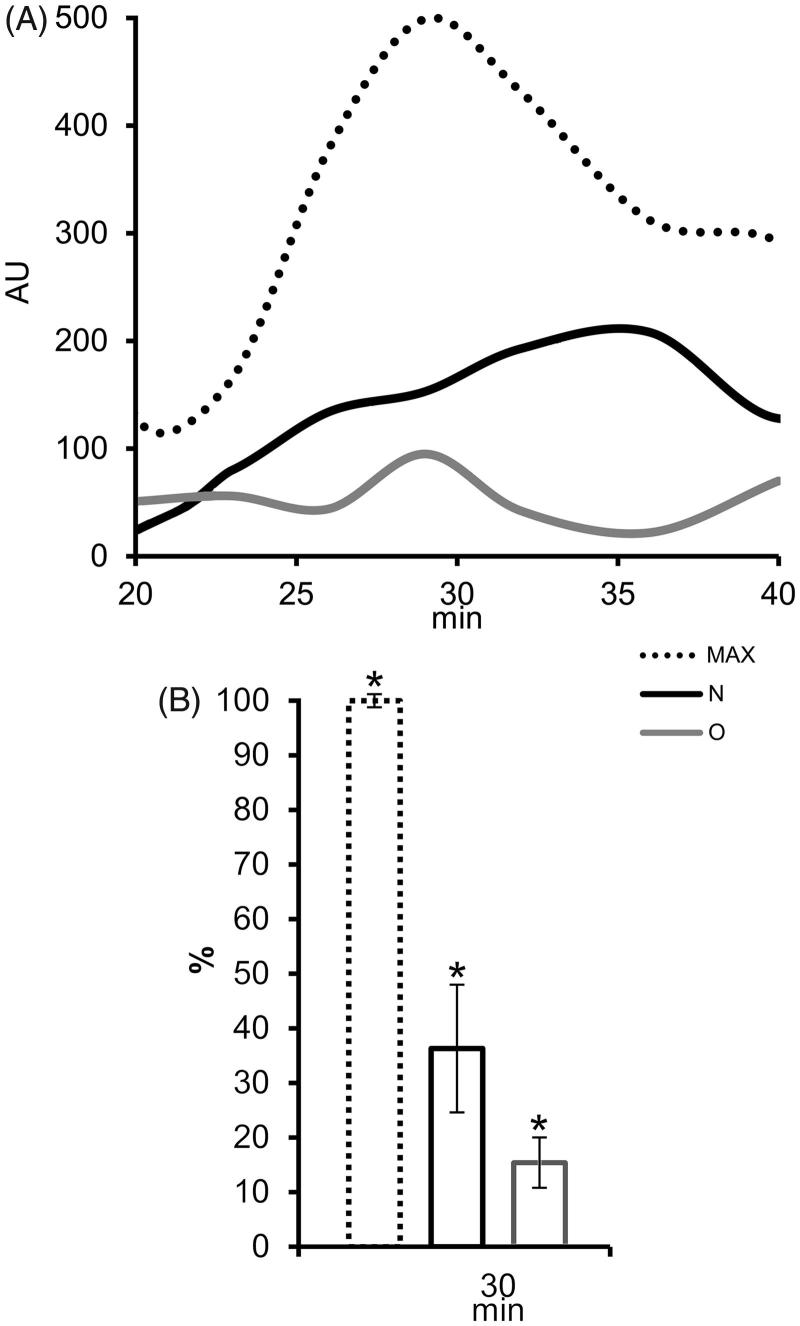
The effect of seminal prostasomes on reactive oxygen species (ROS) production in polymorphonuclear neutrophils (PMN). A: Kinetics of ROS production in PMN after activation with 12-myristate-13-acetate phorbol ester (PMA) alone (MAX) or in the presence of charge-resolved population of N and O, eluted with 1 M NaCl. B: Comparison of ROS production (%) in PMN after 30 min of PMA addition, alone or in the presence of charge-resolved population of N and O, eluted with 1 M NaCl shows statistically significant decrease for all. The results are mean values of three experiments, and vertical bars present percent of standard deviation. Asterisk (*) indicates *P* < 0.05. (AU = arbitrary chemiluminescence units; N = seminal prostasomes from normozoospermic men; O = seminal prostasomes from oligozoospermic men).

The influence of differences in surface composition on known antioxidative activity of prostasomes was checked using an assay on PMN. ROS production increased markedly after stimulation with phorbol myristate acetate, reaching its maximum after 30 min (Figure 5(A)). In the presence of populations of prostasomes of normozoospermic men and those of oligozoospermic men eluted with 1M NaCl, ROS production was lower, but still present (Figure 5(A)). At maximum ROS production time (30 min) this decline was statistically significant for all tested samples (Figure 5(B)).

## Discussion

In agreement with data on disparate SP compositions in normo- and oligozoospermic men (13,23–25), and presumed influence of extracellular environment on EVs, the results obtained indicated distinct differences in surface glycans of related seminal prostasomes. Altered presentation of sialylated and mannosylated structures was clearly observed when corresponding samples were analyzed using TEM, whereas both IEC and total protein patterns were not so decisive/comprehensive.

It is known that EVs comprise a continuum of particles of different size, morphology, and density (1). These parameters have been shown to vary considerably between EV preparations (also influenced by the techniques used), making EV heterogeneity in individual preparations intrinsic and general (26). Since we approached EV surface glycosylation, these parameters were found not relevant for our examination. Consequently, glycosylation was analyzed on prostasomes isolated from pooled SP samples in order to average individual sample heterogeneity, whereas surface glycosylation as revealed by IEC was taken as a reference for comparison.

Thus, when resolved on IEC, prostasomes in both sample groups (also in each of their pools) concentrated in the highest-charge CD63-positive population. It exhibited broad elution profile that indicated two differently abundant subpopulations being in agreement with previous data on their existence (10). ConA-binding patterns which follow SNA-binding patterns in the highest-charge population (prostasome-associated) are assumed to reflect the specificities of the extracellular environment where they are released. Additional heterogeneity, especially in prostasomes from oligozoospermic men, seen as ConA-binding to lower-charge populations (non-prostasomal), could be due to separation of co-isolated proteins/protein complexes, which represent non-specific/common contaminants as shown previously (20). In this study that was supported by their variable presence in all examined SP pools. Indeed, when prostasomal surface glycans were visualized by lectin-TEM, another aspect of IEC-assumed heterogeneity between the examined samples clearly emerged. Subtle differences regarding distribution of SNA-binding sites, but pronounced ones regarding those of ConA, were revealed in prostasomes from normozoospermic compared to prostasomes from oligozoospermic men. SNA, specific for sialylated structures, bound only vesicles (with no respect to sample examined), whereas ConA specific for various mannose-containing sialylated and non-sialylated N-glycans additionally bound to material known as amorphous substance (27,28). This was more pronounced in prostasomes isolated from SP with abnormal semen parameters.

Separation of prostasomes, in the highest-charge IEC fraction, is in accordance with data indicating that they display a net negative surface charge (29). In relation to this, it is known that prostasomes inhibit production of reactive oxygen species by stabilizing the plasma membrane (22,30) and that this effect is expected to rely on surface charge. In general, anionic nanoparticles do not pass through the cell membrane, i.e. they prevent membrane damage/hinder decomposition (31). When differences in surface glycans (associated with negatively charged prostasomes) were examined to discover whether they have implications on the antioxidative capacity of prostasomes from normozoospermic and oligozoospermic men, the results obtained indicated that ROS production in PMN was decreased with no differences between the examined samples. The possibility could not be overlooked that changes in the presentation of surface glycans may have more pronounced effects on other types of known prostasomal activity (32), involving protein–protein or protein–carbohydrate interactions at the level of a particular molecule. In relation to this, gal-3, known as part of the prostasomal proteome (33,34), was found to be present on the surface of vesicles from normozoospermic and oligozoospermic men. Gal-3 is a versatile carbohydrate-binding protein involved in basic physiological processes (35,36). However, regardless of any biological consequence of its possibly altered accessibility to binding partners in the context of differently presented glycans, this may represent an interesting and unexpected regulatory mechanism, in general. It has already been shown that the glycans of EVs contribute in a specific way to the complexity of the normal SP glycome compared to other SP constituents (37). In this study, a further distinction among prostasomes was visualized at the level of particular vesicles, pointing to molecular arrangements/setup at the ultrastructural level as a naturally occurring process and an overlooked source of heterogeneity.
